# Supplementary material for: Characterization of Tellurite Toxicity to Escherichia coli Under Aerobic and Anaerobic Conditions
Source: Int J Mol Sci. 2025 Jul 28;26(15):7287. doi: 10.3390/ijms26157287 (PMC12348037; doi:10.3390/ijms26157287)
Supplement: Supplementary file 1 [file ijms-26-07287-s001.zip › Figure S1.docx]

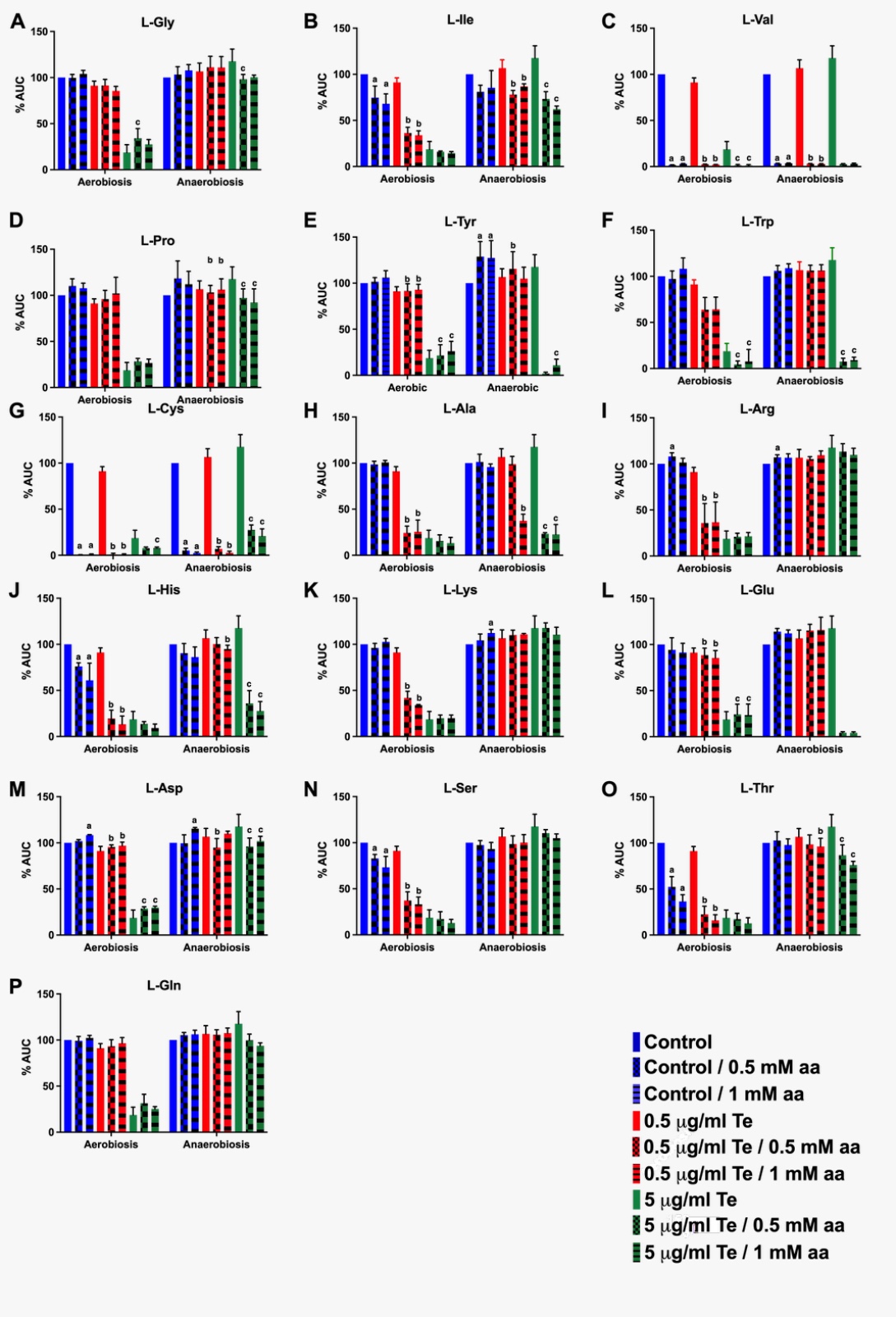


Figure S1. Effect of other amino acids on tellurite toxicity in *E. coli* under aerobic and anaerobic conditions. % AUC values are shown relative to untreated controls for cultures supplemented with 0.2 mM of other amino acids (arginine, tryptophan, cysteine, lysine, histidine, glutamate) with or without tellurite. Statistical significance is shown relative to the corresponding treatment without amino acids: a, *p* < 0.001; b, *p* < 0.01; c, *p* < 0.05.
